# Circulating MicroRNA-223 Serum Levels Do Not Predict Sepsis or Survival in Patients with Critical Illness

**DOI:** 10.1155/2015/384208

**Published:** 2015-02-24

**Authors:** Fabian Benz, Frank Tacke, Mark Luedde, Christian Trautwein, Tom Luedde, Alexander Koch, Christoph Roderburg

**Affiliations:** ^1^Department of Medicine III, University Hospital RWTH Aachen, Pauwelsstrasse 30, 52074 Aachen, Germany; ^2^Department of Cardiology and Angiology, University of Kiel, Schittenhelmstrasse 12, 24105 Kiel, Germany

## Abstract

*Background and Aims*. Dysregulation of miR-223 was recently linked to various diseases associated with systemic inflammatory responses such as type 2 diabetes, cancer, and bacterial infections. However, contradictory results are available on potential alterations of miR-223 serum levels during sepsis. We thus aimed to evaluate the diagnostic and prognostic value of miR-223 serum concentrations in patients with critical illness and sepsis. *Methods*. We used i.v. injection of lipopolysaccharide (LPS) as well as cecal pole ligation and puncture (CLP) for induction of polymicrobial sepsis in mice and measured alterations in serum levels of miR-223. These results from mice were translated into a large and well-characterized cohort of critically ill patients admitted to the medical intensive care unit (ICU). Finally, results from analysis in patients were correlated with clinical data and extensive sets of routine and experimental biomarkers. *Results*. Although LPS injection induced moderately elevated serum miR-223 levels in mice, no significant alterations in miR-223 serum levels were found in mice after CLP-induced sepsis. In accordance with these results from animal models, serum miR-223 levels did not differ between critically ill patients and healthy controls. However, ICU patients with more severe disease (APACHE-II score) showed moderately reduced circulating miR-223. Strikingly, no differences in miR-223 levels were found in critically ill patients with or without sepsis, and serum levels of miR-223 did not correlate with classical markers of inflammation or bacterial infection. Finally, low miR-223 serum levels were moderately associated with an unfavorable prognosis of patients during the ICU treatment but did not predict long-term mortality. *Conclusion*. Recent reports on alterations in miR-223 serum levels during sepsis revealed contradictory results, preventing a potential use of this miRNA in clinical routine. We clearly show that miR-223 serum levels do not reflect the presence of sepsis neither in mouse models nor in a large cohort of ICU patients and do not indicate clinical outcome of critically ill patients. Thus miR-223 serum levels should not be used as a biomarker in this setting.

## 1. Introduction

Sepsis represents a major cause of death for critically ill patients during intensive care unit (ICU) treatment [1]. In this setting, early diagnosis and initiation of specific therapeutic measures were shown to considerably reduce mortality in critically ill patients with sepsis. Thus, in addition to established laboratory parameters and clinical scores, novel biomarkers may significantly improve treatment and prognosis assessment in patients admitted to the intensive care unit [2].

MicroRNAs (miRNAs) represent small RNAs of 22 nucleotides in length that do not withhold the sequential information to transcribe for proteins but function as critical regulators of gene expression in eukaryotes [3, 4]. miRNAs are involved in various pathophysiological processes such as cell injury, proliferation, or carcinogenesis [5–7]. A specific deregulation of microRNA miR-223 was described in different disease states associated with a systemic inflammatory response such as bacterial infections or autoimmune diseases. Although this may represent an epiphenomenon of these diseases, recent evidence suggests that miR-223 is actively involved in regulation of inflammatory processes by limiting the inflammation to prevent collateral damage and tissue injury [8]. In line with this assumption various targets of the miR-223 belong to the superfamily of inflammatory genes such as granzyme B, IKK-alpha, and STAT3. Moreover, the expression of miR-223 itself is regulated by inflammatory pathways such as NF-*κ*B or the TLR4 pathway, suggesting a deep integration of this miRNA in inflammatory signaling cascades driving inflammation and anti-infectious responses in general [8].

Besides their functional involvement in gene expression, miRNAs can be released to the extracellular compartment and are easily detectable in body fluids such as blood, sweat, and urine. However, at present little is known about miRNAs deregulated in the serum of patients with critical illness and sepsis. It was demonstrated that serum levels of different miRNAs such as miR-133a, miR-150, and miR-146a are significantly altered in critically ill patients compared to healthy controls [9–11]. Importantly, alterations in miR-223 serum levels were described in patients with sepsis, but have yet led to conflicting results. While Wang et al. described higher levels of miR-223 in septic patients [12] and suggest a direct association between high miR-223 serum levels and patients' outcome [13], Wang et al. reported lower levels of miR-223 in the serum of patients with septic disease [11], thus highlighting the need for further studies clarifying the regulation of miR-223 during bacterial infection and sepsis.

Considering the complexity of septic disease in humans, we first analysed serum levels of miR-223 in different well-established models of experimental polymicrobial sepsis in mice such as cecal ligation and puncture (CLP) surgery and lipopolysaccharide (LPS) injection. To translate our findings from these animal models to human pathogenesis, we analyzed miR-223 serum levels in a large, well-characterized cohort of 221 critically ill patients (with and without sepsis), demonstrating that serum levels of miR-223 do not reflect the presence of septic disease and are not associated with the clinical outcome of patients during intensive care unit treatment.

## 2. Materials and Methods

### 2.1. Study Design

In this present study, we prospectively recruited 221 patients (141 male, 80 females) with a median age of 63 years (range: 18 to 89 years) that were consecutively admitted to the General Internal Medicine Intensive Care Unit (ICU) at the University Hospital Aachen [14]. Blood samples were collected upon admission to the ICU (prior to therapeutic interventions), centrifuged for 10 min at 2000 g, and serum samples were stored at −80°C until use. In addition, 75 healthy blood donors (47 male, 29 female, median age 33 years, and range 18–67) with normal values for blood counts, C-reactive protein, and liver enzymes were recruited from the local blood donation center as healthy controls [14]. Patients were included in the study upon providing written informed consent and the ethics committees approved this consent procedure. The study protocol is in line with Declaration of Helsinki and was approved by the local ethics committee (Ethics Committee of the University Hospital Aachen, RWTH University, Aachen, Germany, reference number EK 150/06). We did not include patients who were expected to have a short-term (<72 h) intensive care treatment due to postinterventional observation or acute intoxication into this study [14]. Patients who fulfilled the criteria proposed by the American College of Chest Physicians and the Society of Critical Care Medicine Consensus Conference Committee for severe sepsis and septic shock were categorized as sepsis patients and the others as nonsepsis patients. All patients were treated in accordance with current guidelines for treatment of sepsis (Surviving Sepsis Campaign) and specific guidelines of the respective boards. The clinical course of patients was followed up for a period of three years by directly contacting the patients, the patients' relatives, or their primary care physician [10].

### 2.2. Patient Characteristics

The criteria of bacterial sepsis at the time point of admission to the ICU were fulfilled by 137 of the 221 patients (Table 1) and pneumonia represented the most common site of infection (Table 2). Sepsis patients were more often and for longer terms in need of mechanical ventilation and had significantly higher levels of routinely used biomarkers of inflammation (C-reactive protein, procalcitonin, and white blood cell count) as compared to the nonsepsis patients' cohort (Table 1). In nonsepsis patients, cardiopulmonary diseases (myocardial infarction, pulmonary embolism, and cardiac pulmonary edema), decompensated liver cirrhosis, or other critical conditions represented the predominant etiologies, which did not differ in age or sex from sepsis patients. Both groups did not differ in Acute Physiology and Chronic Health Evaluation (APACHE) II and Simplified Acute Physiology Score 2 (SAPS2) score, vasopressor demand, or laboratory parameters indicating liver or renal dysfunction (Table 1).

### 2.3. Cecal Ligation and Puncture (CLP)

The mouse model of cecal ligation and puncture (CLP) as a well-established model for polymicrobial sepsis was used. Male C57Bl/6 mice (*n* = 14, 6–8 wk of age) were purchased from The Jackson Laboratory (Bar Harbor, ME) and were subjected to CLP surgery, as described previously [15]. Blood was taken before and 24 h after surgery and serum was stored in −80°C until use. Animals received humane care according to European, national, and institutional regulations.

### 2.4. LPS Injection

Endotoxin mediated sepsis was induced by lipopolysaccharide (LPS), which is a component of Gram-negative bacteria. 2.5 *μ*g per gram bodyweight was injected intraperitoneally in male C57BL/6 mice, and mice were killed 8 hours later.

### 2.5. miRNA Isolation from Serum

For miRNA isolation we used 400 *μ*L serum from human or 70 *μ*L serum from mice. As described previously [16], serum was spiked with miScript miRNA Mimic SV40 (Qiagen 2 *μ*M, 1 *μ*L/100 *μ*L serum) for sample normalization. 800 *μ*L phenol (Qiazol) and 200 *μ*L chloroform were added to the sample and mixed vigorously for 15 sec followed by incubation at room temperature for 10 min. Then samples were centrifuged for 15 min at 12,000 g and the aqueous phase, containing total RNA, was precipitated with 500 *μ*L 100% isopropanol and 2 *μ*L glycogen (Fermentas, St. Leon-Rot, Germany) overnight at −20°C. After centrifugation at 4°C for 30 min (12,000 g) the pellets were washed once with 70% ethanol and precipitated RNA was resuspended in 30 *μ*L RNase-free water (Ambion, Austin, TX) [9].

### 2.6. miRNA Isolation from Tissue

As described previously [16] total RNA was purified from tissue using Trizol reagent (Invitrogen, Carlsbad, CA, USA) and RNeasy Mini kit (Qiagen, Hilden, Germany). The quantity and quality of the RNA were determined spectroscopically using a nanodrop (ThermoScientific, Waltham, MA, USA).

### 2.7. Quantitative Real-Time PCR

Relative expression of miR-223 in serum and tissue was measured with quantitative real-time PCR (qPCR) as recently described [16]. In detail, total RNA (1 *μ*g for tissue RNA, 5 *μ*L for serum RNA) was used to synthesize cDNA utilizing miScript Reverse Transcriptase Kit (Qiagen) according to the manufacturer's protocol and was then resuspended in suitable amounts of H_2_O. cDNA samples (2 *μ*L) were used for qPCR in a total volume of 25 *μ*L using the miScript SYBR Green PCR Kit (Qiagen) and miRNA-specific primers (Qiagen, sequence: 5′UGUCAGUUUGUCAAAUACCCCA) on a qPCR machine (Applied Biosystems 7300 Sequence Detection System, Applied Biosystems, Foster City, CA). All real-time PCR reactions were performed in duplicate. Data were analyzed using the SDS 2.3 and RQ Manager 1.2 software packages and relative gene expression was generated using the 2^−ΔΔCT^ method (ΔCT target gene −ΔCT control gene).

### 2.8. Statistical Analysis

All statistical analyses were performed with SPSS 20 (IBM SPSS Statistics) as recently described [9]. Data are displayed as median and range considering the skewed distribution of most parameters. Gaussian distribution was tested with Kolmogorov-Smirnov test. Differences between two groups were assessed by Student's *t*-test or Wilcoxon rank-sum test and multiple comparisons between more than two groups have been conducted by ANOVA with Bonferroni's test or Dunn's test for post hoc analysis. Box-whisker-plot graphics illustrate a statistical summary. Here the box represents the median with interquartile range (IQR) and the “whiskers” include all values smaller than the upper quartile plus 1.5 ∗ IQR and larger than the lower quartile minus 1.5 ∗ IQR. Values outside of the whiskers are displayed as separate points and represent outliers. All values, including outliers, have been included for statistical analyses. Correlations between variables have been analysed using the Spearman correlation test. Receiver operating characteristic (ROC) curve analysis and the derived area under the curve (AUC) statistic provide a global and standardized appreciation of the accuracy of a marker or a composite score for predicting an event. ROC curves were generated by plotting sensitivity against 1 − specificity. Kaplan-Meier curves were plotted to display the impact on survival and between-group differences were assessed using the log-rank test. Cox regressions were used to identify factors predicting ICU mortality or overall mortality. All reported *P* values were two-tailed and a *P* value less than 0.05 was considered to indicate statistical significance.

## 3. Results 

### 3.1. miR-223 Serum Levels in Murine Models of Septic Diseases

**Figure 1:**
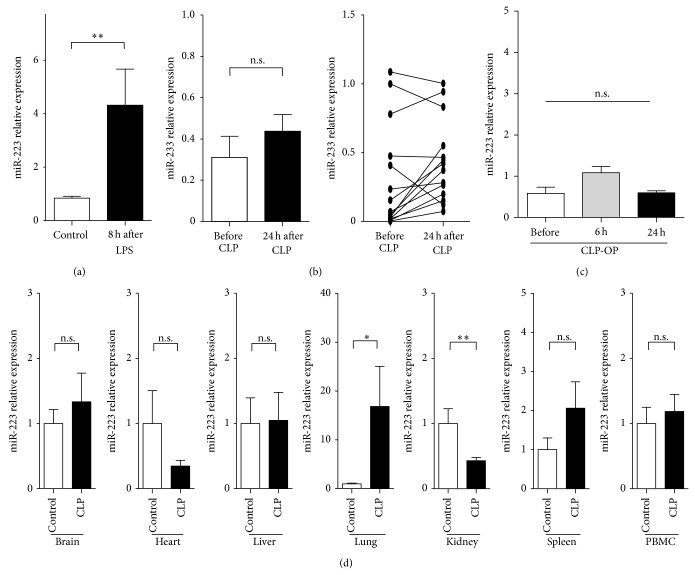
miR-223 serum levels in murine models of septic diseases. (a) Serum was obtained from C57Bl/6j wild-type mice at baseline and 8 hours after injection of 2.5 *μ*g per gram body weight. miR-223 serum levels were measured. The bar graphs represent mean ± SEM from *n* = 5 animals per group. (b and c) Serum was obtained from C57Bl/6j wild-type mice at baseline and 8 hours and 24 hours after induction of polymicrobial sepsis by CLP. miR-223 serum levels were measured. The bar graphs represent mean ± SEM; the line graphs display paired pre- and postoperative values for individual animals (*n* = 14). (d) Relative expression of miR-233 in different organs in mice after SHAM or CLP surgery (kidney, spleen, liver, lung, heart, brain, and peripheral blood mononuclear cells (PBMC)) from C57Bl/6j wild-type mice.

Based on the contradictory data on alterations of miR-223 serum levels in patients with sepsis we decided to first analyse miR-223 serum concentrations in highly standardized mouse models of septic disease. Therefore, CLP procedures and LPS injections were performed in C57Bl/6 mice. As determined by miRNA-specific qPCR, miR-223 levels were moderately, but significantly, elevated 8 h after injection of LPS (Figure 1(a)). In contrast, levels of circulating miR-223 remained unaffected at different time points after induction of sepsis by using the CLP model, which closely resembles human sepsis (Figures 1(b) and 1(c)). Finally, miR-223 expression was analysed in different organs after induction of sepsis by CLP surgery to further determine potential mechanism regulating miR-223 serum levels in sepsis. These analyses revealed a significant upregulation in the lung, while expression of miR-223 was downregulated in kidney and unaffected from the induction of septic disease in the other organs, including liver, brain, heart, and muscle (Figure 1(d)).

### 3.2. miR-223 Serum Levels in Critically Ill Patients and Healthy Controls

**Figure 2:**
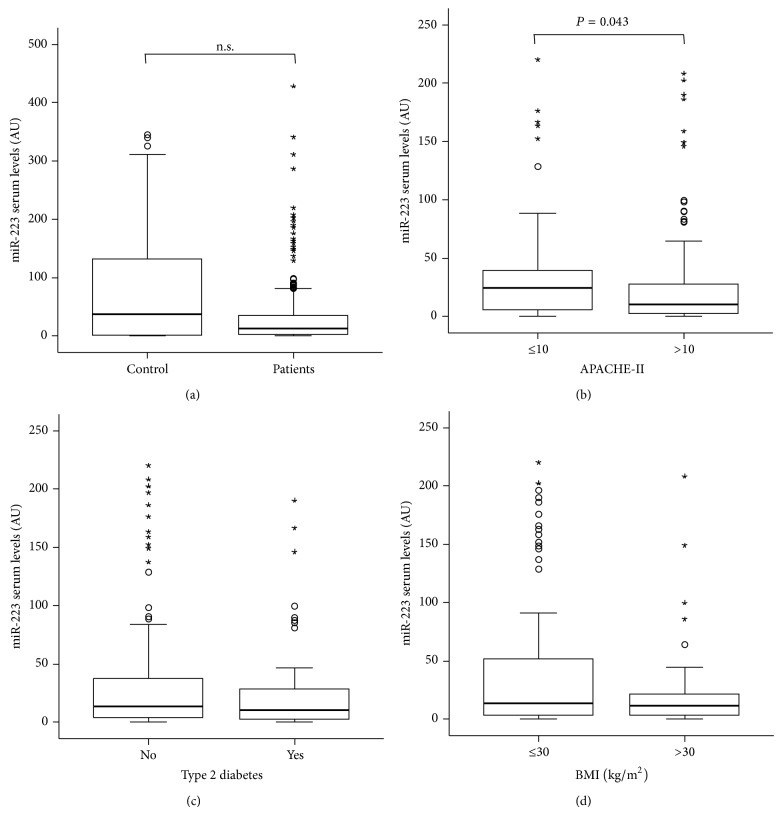
miR-223 serum levels in critically ill patients and healthy controls. (a) Serum concentrations of miR-223 were determined by qPCR in RNA extracts from serum of patients at admission to the intensive care unit (ICU). miR-223 levels were unchanged in critically ill patients (*n* = 221) compared with healthy controls (*n* = 75). (b) Serum miR-223 concentrations at admission to the ICU were significantly changed in critically ill patients with high initial Acute Physiology and Chronic Health Evaluation (APACHE) II scores (>10) in comparison to patients with low APACHE-II scores (≤10). (c) Serum miR-223 concentrations at admission to the ICU are unchanged in patients with or without type 2 diabetes mellitus. (d) Serum miR-223 levels at admission to the medical ICU are independent of the presence of obesity in critically ill patients. Box plots are displayed, where the bold line indicates the median per group, the box represents 50% of the values, and the horizontal lines show minimum and maximum values of the calculated nonoutlier values; asterisks and open circles indicate outlier values.

We next analyzed levels of circulating miR-223 in sera of 221 patients at admission to the ICU as well as in 75 healthy volunteers. miR-223 concentrations were slightly lower in critically ill patients compared to healthy controls (*P* = 0.141; Figure 2(a)). When we analyzed serum levels of miR-223 with respect to disease severity, we found significantly lower levels in patients with more severe disease according to higher APACHE-II scores (>10), compared to patients with lower APACHE-II scores (<10) (*P* = 0.043; Figure 2(b)).

The metabolic status of patients was shown to influence the outcome of critically ill patients [17]. Since miR-223 was shown to be involved in the pathophysiology of type 2 diabetes [8], we analyzed potential correlations between miR-223 serum levels and the presence of obesity or type 2 diabetes. Importantly, we found no significant differences in circulating miR-223 between patients with obesity or normal body weight (Figure 2(c)) and those with or without type 2 diabetes mellitus, respectively (Figure 2(d)). In addition, no differences were found when patients were compared by their age or gender (data not shown).

### 3.3. miR-223 Serum Levels Do Not Indicate Sepsis in Critically Ill Patients

**Figure 3:**
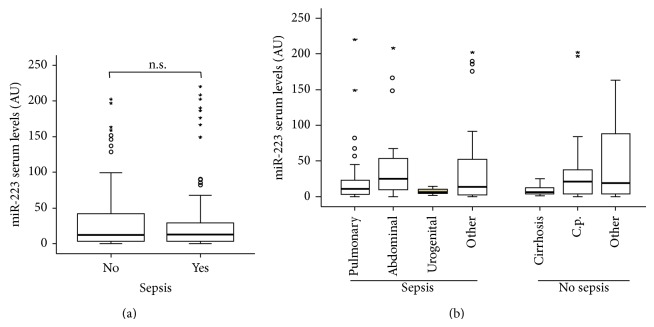
miR-223 serum levels do not indicate sepsis in critically ill patients. (a) Serum levels of miR-223 were not different in patients that fulfilled sepsis criteria (*n* = 157) compared to patients with nonseptic etiology of critical illness. (b) miR-223 serum levels did not vary between the different etiologies of septic or nonseptic disease.

Elevated levels of miR-223 were suggested to discriminate between SIRS and sepsis patients with high sensitivity and specificity [11]. Our cohort of ICU patients featured both patients with sepsis (*n* = 137) and patients that did not fulfill sepsis criteria (*n* = 84). Thus, we further investigated the impact of sepsis on miR-223 serum concentrations in our cohort. In these analyses, no significant differences in miR-223 levels between septic and nonseptic patients were evident (*P* = 0.529; Figure 3(a)). Of note, the fact that circulating miR-223 is independent of the presence of sepsis was further substantiated by correlation analyses revealing that serum miR-223 levels were not correlated to established markers of systemic inflammation and bacterial infection such as C-reactive protein (CRP), procalcitonin (PCT), interleukin-6 (IL-6), interleukin-10 (IL-10), or tumor necrosis factor (TNF) in critically ill patients (Table 3).

In order to investigate the impact of the underlying etiology of sepsis/critical illness on miR-223 serum levels more precisely, we again performed subgroup analyses. The cohort of sepsis patients was subdivided into a pulmonary and a nonpulmonary site of infection and the nonsepsis patients were categorized into liver cirrhosis, cardiovascular disorders, and others. However, also this analysis revealed no differences in miR-223 serum concentration between the different subgroups, thus excluding the fact that potential alterations in miR-223 levels might only exist in a specific group of patients and be masked if these patients are merged with other patients (Figure 3(b), Table 2).

In summary, our analysis reveals that, in contrast to previous studies reporting a downregulation [11] or upregulation [12] of miR-223 in serum of patients with septic disease, miR-223 levels are only slightly altered in critical illness and sepsis, indicating that miR-223 measurements from serum are not suitable to detect sepsis.

### 3.4. miR-223 Serum Concentrations Do Not Predict Survival in Critically Ill Patients

Multiple organ failure represents a fearful complication of sepsis and sepsis shock syndrome, often leading to death in critically ill patients. To determine whether miR-223 serum levels might be indicative for patients' prognosis during and after ICU treatment, we first performed correlation analysis between miR-223 levels and classical markers of organ dysfunction. While miR-223 serum levels showed a significant correlation with decreased renal function (Table 3), no correlation with acute liver injury or an impaired liver synthesis capacity could be established. Moreover, we found no correlation to classical prognosis scores or other parameters indicating patients' outcome on ICU treatment such as ventilation time or time spent on ICU treatment (Table 3).

**Figure 4:**
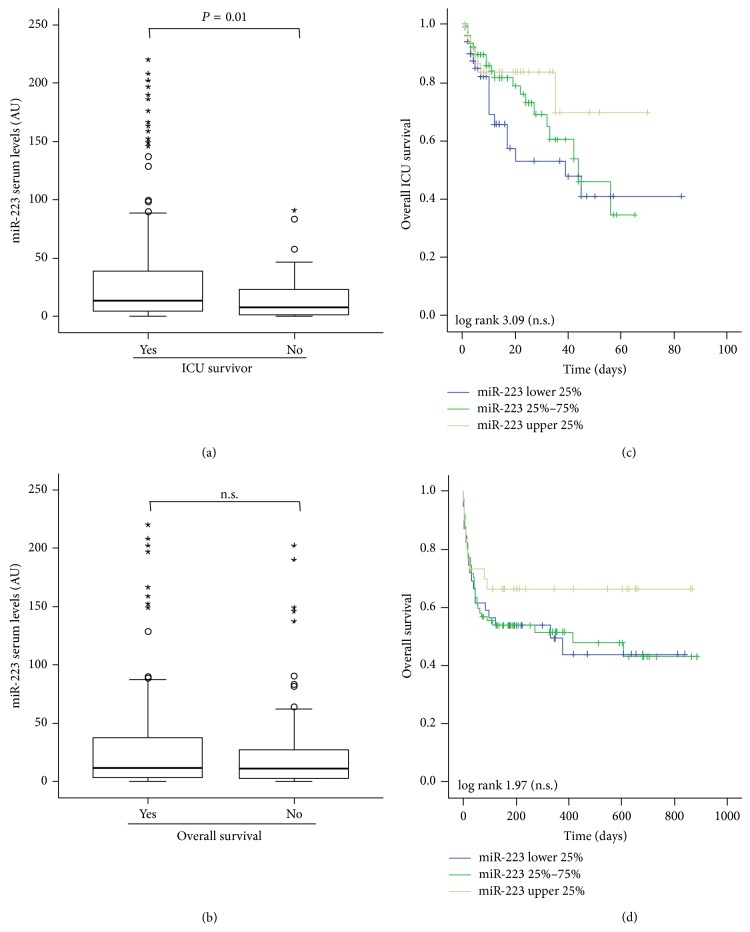
miR-223 serum concentrations do not predict survival in critically ill patients. (a) Patients that died during the course of ICU treatment had lower miR-223 serum levels on admittance to ICU compared to survivors (*P* = 0.010, *U*-test). (b) Kaplan-Meier survival curves of ICU patients are displayed, showing that miR-223 serum levels had no significant (Cox regression) influence on ICU or long-term survival in critically ill patients. (c) Patients that died during long-term follow-up had similar miR-223 serum concentration on admittance to ICU compared to surviving patients (*P* = 0.386, *U*-test). (d) Kaplan-Meier survival curves of ICU patients are displayed, showing that miR-223 serum levels had no influence on long-term survival in critically ill patients. In (a) and (b) box plots are displayed, where the bold line indicates the median per group, the box represents 50% of the values, and the horizontal lines show minimum and maximum values of the calculated nonoutlier values.

Despite these negative correlation analyses and the fact that miR-223 serum levels did not significantly vary between critically ill patients and healthy controls or between septic and nonseptic patients, we next analyzed whether they might be useful in predicting mortality in critically ill patients. In line with the reduced miR-223 concentrations in patients with high APACHE-II scores (see Figure 2(b)), patients that died during the ICU treatment showed lower levels of miR-223 levels compared to survivors, supporting a role for miR-223 in estimating patients' outcome during ICU treatment (*P* = 0.010; Figure 4(a)). Kaplan-Meier curve analysis revealed that patients with lower miR-223 levels (e.g., of the lower quartile) demonstrated an impaired ICU survival compared to patients with miR-223 concentrations within the upper quartile of all patients; however these differences failed to reach statistical significance in Cox regression analyses.

In our cohort of critically ill patients, 49 died on the ICU while additional 45 patients died after release from ICU. In contrast to the data on ICU survival, patients that died during long-term follow-up showed no differences in miR-223 levels compared to survivors (*P* = 0.386; Figure 4(c)). In line with that, Kaplan-Meier curve analysis revealed no role for miR-223 serum measurements in determining patients' long-term prognosis (Figure 4(d)), thus arguing against a strong role of miR-223 as a blood based marker for prediction of critically ill patients' prognosis.

## 4. Discussion

Despite enormous advances in diagnosis modalities, triaging patients at the emergency room for relocation to the ICU or guiding therapeutic decisions within the first week of ICU treatment represents a major challenge in the treatment of critically ill patients. However, such decisions are of tremendous relevance for critically ill patients [18]. In this setting, markers allowing decision about patients' treatment and clinical course may be of significant benefit. Various authors demonstrated that commonly used markers like CRP and PCT might represent diagnostic and prognostic biomarkers in this setting, especially when used in combination with clinical severity scores or multimarker approaches [19, 20]. Besides CRP and PCT a variety of different protein-based markers such as suPAR, inducible protein 10 (IP10), neutrophil gelatinase-associated lipocalin (NGAL), natriuretic peptides, mature adrenomedullin (ADM), and thrombopoietin was tested; however none of these experimental markers could be translated into clinical use [14, 21]. Several authors therefore speculated that novel, for example, miRNA-based markers might perform better and could therefore enter clinical routine.

miRNAs have recently been associated with the pathogenesis of systemic inflammation and infection [22]. Beside others, it was shown that miR-223 is critically involved in the differentiation and maturation of key players of the innate immune response. An increased immune response towards infectious agents such as* Candida albicans* was shown in miR-223 mutant mice (miR-223^−/Y^). Moreover, in endotoxin-challenge models, miR-223^−/Y^ mice demonstrated elevated tissue destruction, thus highlighting a potential role of miR-223 in the pathophysiology of septic diseases and providing evidence for analysis of miR-223 in serum of sepsis patients.

miR-223 has recently been proposed as a novel serum biomarker for sepsis and septic shock disease in two different Asian populations of sepsis patients. Of note, results from these studies were conflicting: on the one hand, it was demonstrated in a cohort of 116 patients (43 with mild sepsis and 73 with severe sepsis/septic shock) that elevated miR-223 levels are indicative of the presence of septic disease [12] and correlate with an impaired prognosis in these patients [13]. On the other hand, in a cohort of 80 patients (30 with SIRS and 50 with sepsis) miR-223 levels were decreased in patients that fulfilled criteria of sepsis compared to healthy controls [11]. In the present study, we analysed miR-223 serum levels in a well-defined cohort of 223 critically ill patients. However, despite the large number of samples analysed, we failed to demonstrate significant alterations in serum miR-223 concentrations in critical illness and sepsis.

The conflicting results of previously published results and our current findings might partly be explained by differences of experimental procedures in the different studies. While we used spiked-in RNA (SV40) for normalization of miR-223 serum levels, Wang et al. used an internal reference gene, namely, snU6, for normalization in their studies [12, 13]. We and other groups recently demonstrated that snU6 might be regulated itself in inflammatory diseases, potentially affecting these previous results. Of note, we found a trend towards lower levels of miR-223 in critical illness, which is in line with the data of Wang et al. [11] who also used spiked-in RNA for normalization. Moreover, the differences between the different studies might also be related to the size and characteristics of the patient cohorts analyzed in the different studies. We report data from a large consecutively recruited cohort that covered a broad spectrum of critically ill patients with regard to severity of illness as reflected by APACHE-II and SOFA scores. Of note, besides sepsis, conflicting results of miR-223 serum levels have also been described in patients with HCC or chronic hepatitis B [23–25], highlighting the need for further efforts in defining standards for sample preparation, data normalization, and data analysis in this setting.

We recently found a concordant regulation of miR-133a in the serum of patients with sepsis and mice after induction of septic disease [9]. In the present study we demonstrate that miR-223 levels remained unchanged in mice after CLP surgery, thus reflecting the situation in patients. In contrast to the results from CLP procedure, we found elevated serum levels of miR-223 in mice after LPS treatment, which is in line with previous reports. These on the first view contradictory results might be explained by differences between the two models: LPS administration induces systemic inflammation that mimics some of the initial clinical features of sepsis (such as increases in proinflammatory cytokines) but does not feature bacteremia, representing the sepsis defining event in human disease [26–28]. Moreover, LPS causes much earlier and higher peak levels of cytokine expression compared with levels observed in human sepsis [27, 29, 30]. Consequently, LPS-induced murine sepsis fails to mimic the different immunological stages of human sepsis: a proinflammatory phase and a compensatory anti-inflammatory phase. In contrast, CLP-induced sepsis increased lymphocyte apoptosis, which mimics immunosuppression at the later phase of human sepsis [31–33]. In this respect, CLP-induced sepsis is completely different from LPS-induced sepsis and more closely mimics human sepsis [34, 35].

In summary, miR-223 serum levels of critically ill and sepsis patients are not significantly regulated compared to healthy controls and are only modestly associated with disease severity or outcome. Our data thus strongly argue against a potential use of miR-223 as a blood based biomarker for septic disease.

## Figures and Tables

**Table 1:** Baseline patient characteristics.

Parameter	All patients	Nonsepsis	Sepsis	*P* value
Number	221	84	137	n.a.
Sex (male/female)	141/80	56/28	85/52	n.s.
Age median (range) [years]	63 (18–89)	62 (18–85)	64 (20–89)	n.s.
APACHE-II score median (range)	17 (2–40)	15 (2–33)	18.0 (3–40)	n.s
SAPS2 score median (range)	42.5 (0–79)	41 (13–72)	43 (0–79)	n.s.
ICU days median (range)	7 (1–83)	5 (1–45)	10 (1–83)	<0.001
Death during ICU [%]	22.2	13.1	27.7	<0.05
Ventilation [%]	67.1	60.5	71.2	<0.01
Body mass index	26.08 (16.6–86.5)	26.1 (16.6–53.3)	26.1 (18.3–86.5)	n.s.
Creatinine	1.3 (0–15)	1.0 (0.3–15.0)	1.5 (0.0–10.7)	n.s.
Albumin	27.0 (15.2–52.2)	29.2 (0.0–52.2)	25.8 (0.0–41.0)	n.s.
WBC median (range) [×10³/*μ*L]	12.2 (0.1–67.4)	11.6 (1.8–27.7)	12.7 (0.1–67.4)	n.s.
CRP median (range) [mg/dL]	94.0 (<5–230)	17 (5–230)	165 (<5–230)	<0.001
Procalcitonin median (range) [*μ*g/L]	0.7 (0–180.6)	0.2 (0.1–100.0)	2.3 (0.0–180.6)	<0.001
Interleukin-6 median (range) [pg/mL]	105 (0–83000)	63 (4–83000)	220 (0–28000)	<0.001
Tumor necrosis factor median [pg/mL]	19 (4.9–140)	16.5 (8.0–100)	23.5 (4.9–140)	<0.001
INR	1.18 (0–9.2)	1.16 (0.90–4.32	1.18 (0.00–9.2)	n.s.

APACHE, Acute Physiology and Chronic Health Evaluation; CRP, C-reactive protein; ICU, intensive care unit; INR, international normalized ratio; SAPS, simplified acute physiology score; WBC, white blood cell count.

**Table 2:** Disease etiology of the study population.

	Sepsis	Nonsepsis
	*n* = 137	*n* = 84
Sepsis critical illness (*n* (%))		
Source of infection		
Pulmonary	74 (54.0%)	
Abdominal	28 (20.4%)	
Urogenital	3 (2.2%)	
Other	32 (23.4%)	
Nonsepsis critical illness (*n* (%))		
Cardiopulmonary disease		30 (35.7%)
Decompensated liver cirrhosis		12 (14.3%)
Nonsepsis other		42 (50.0%)

**Table 3:** Correlations of miR-223 serum levels at ICU admission with other laboratory markers.

Parameter	ICU patients	
	*R*	*P*
Markers of liver function		
Cholinesterase	0.135	n.s.
Protein	0.110	n.s.
Albumin	0.089	n.s.
GGT	0.084	n.s.
GLDH	0.116	n.s.
AP	−0.074	n.s.
INR	−0.028	n.s.
Bilirubin total	−0.014	n.s.
Bilirubin direct	0.059	n.s.
AST	0.091	n.s.
ALT	0.167	0.013
Markers of inflammation		
C-reactive protein	−0.010	n.s.
Procalcitonin	−0.085	n.s.
IL-6	0.171	n.s.
IL-10	0.118	n.s.
TNF-alpha	−0.152	n.s.
Amylase	0.218	n.s.
Lipase	0.111	n.s.
Leucocyte counts	0.155	0.021
Markers of renal function		
Creatinine	−0.391	<0.001
cystatin C	−0.384	<0.001
cystatin C GFR	0.379	<0.001
Urea	−0.435	<0.001
Others variables		
Lactate	−0.146	0.031
NT-proCNP	−0.432	<0.001
suPAR	−0.187	0.022
Clinical scoring		
APACHE-II	−0.139	n.s.
SOFA	−0.187	0.030
ICU days	−0.051	n.s.
Days alive	−0.190	n.s.

*R*, correlation coefficient; *P*, *P* value; *R* and *P* values by Spearman rank correlation; INR, international normalized ratio; IL-6, interleukin-6; IL-10, interleukin-10; TNF, tumour necrosis factor; APACHE-II, Acute Physiology and Chronic Health Evaluation II; SOFA, Sequential Organ Failure Assessment Score.
